# Ca_2_Te_3_O_8_, a new phase in the CaO–TeO_2_ system

**DOI:** 10.1107/S2056989018017310

**Published:** 2019-01-01

**Authors:** Matthias Weil

**Affiliations:** a Institute for Chemical Technologies and Analytics, Division of Structural Chemistry, TU Wien, Getreidemarkt 9/164-SC, A-1060 Vienna, Austria

**Keywords:** crystal structure, oxotellurate(IV), calcium tellurite, isotypism, compstru

## Abstract

Single crystals of dicalcium octa­oxidotritellurate(IV), Ca_2_Te_3_O_8_, were obtained from a CsCl/NaCl melt with CaO and TeO_2_ as educts in the molar ratio of 1:2. Ca_2_Te_3_O_8_ crystallizes isotypically with Pb_3_Te_2_O_8_ and is comprised of two unique Ca, four Te and eight O sites. One calcium cation has eight and the other nine coordination partners. Both coordination polyhedra are considerably distorted. Two kinds of oxotellurate(IV) anions with the same formula [Te_3_O_8_]^4−^ are present. One is an infinite zigzag chain anion consisting of pairs of [TeO_4_] bis­phenoids linked to a trigonal–pyramidal [TeO_3_] group with a connectivity of [(TeO_1/1_O_2/2_)(TeO_2/1_O_2/2_)_2_]_*n*_, while the other is a finite anion made up of one central [TeO_4_] bis­phenoid linked to two [TeO_3_] trigonal pyramids and has a connectivity of [(TeO_2/1_O_1/2_)_2_(TeO_2/2_O_2/1_)]. In the crystal, the anions are organized in layers extending parallel to (100). Adjacent layers are held together by the calcium cations to define a three-dimensional framework structure.

## Abstract 2

The crystal structure of Ca_2_Te_3_O_8_ is isotypic with Pb_2_Te_3_O_8_.

## Chemical context   

A partial phase diagram for the pseudo-binary system CaO–TeO_2_ has been determined for the composition range 50–100 mol% TeO_2_ to contain the 1:1 phase CaTeO_3_ and the 1:2 phase CaTe_2_O_5_ (Mishra *et al.*, 1998 ▸). Another phase not reported during the original study of Mishra *et al.* (1998 ▸) is the 4:5 phase Ca_4_Te_5_O_14_, for which full structural details were determined for the normal-pressure and high-pressure forms (Weil, 2004 ▸; Weil *et al.*, 2016 ▸). For compositions CaTeO_3_ and CaTe_2_O_5_, polymorphism was reported on the basis of differential thermal analysis and temperature-dependent X-ray diffracion (Mishra *et al.*, 1998 ▸; Tripathi *et al.*, 2001 ▸), however, without structural details of the corresponding phases. Whereas crystal structure determinations were subsequently performed for four polymorphic forms of CaTeO_3_ (Stöger *et al.*, 2009 ▸; Poupon *et al.*, 2015 ▸), our present knowledge of the CaTe_2_O_5_ structures is restricted to only one form (Weil & Stöger, 2008 ▸; Barrier *et al.*, 2009 ▸) that is not related to the mica-like CaTe_2_O_5_ phase reported nearly 50 years ago (Redman *et al.*, 1970 ▸). In an attempt to grow single crystals of the latter from a salt melt at comparatively low temperatures, a heretofore unknown phase in the CaO–TeO_2_ system was obtained, *viz.* the 2:3 phase Ca_2_Te_3_O_8_.

In this article, preparation conditions, crystal structure and the relation to the isotypic lead(II) analogue Pb_2_Te_3_O_8_ (Champarnaud-Mesjard *et al.*, 2001 ▸) are reported.

## Structural commentary   

The asymmetric unit of Ca_2_Te_3_O_8_ comprises two Ca sites, four Te sites and eight O sites. One Ca site (Ca2) is located on Wyckoff position 8*g* (site symmetry ..*m*), sites Te1 on 4*c* (*m*2*m*), Te2 on 8*f* (*m*..), Te4 on 8*g*, O1 on 8*f*, O2 on 8*e* (2..) and O7 and O8 both on 8*g*; all other sites are on general positions 16*h*.

**Figure 1:**
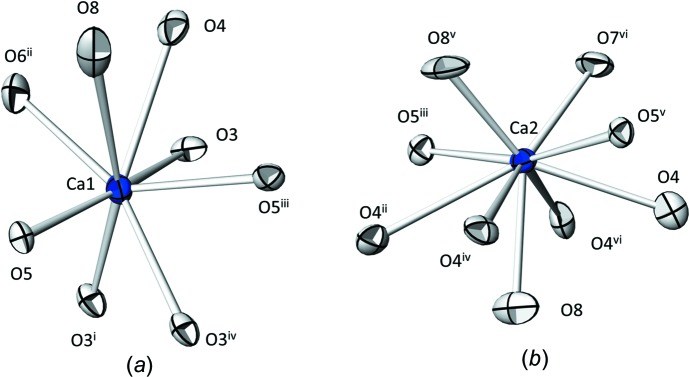
(*a*) The [Ca1O_8_] and (*b*) the [Ca1O_9_] polyhedra in the crystal structure of Ca_2_Te_3_O_8_. Displacement ellipsoids are drawn at the 90% probability level. Symmetry codes refer to Table 1 ▸.

The two Ca^2+^ cations are surrounded by eight (Ca1) and nine (Ca2) O atoms, considering a cut-off value of 3.1 Å for relevant Ca—O distances (Table 1 ▸). The bond valence sums (Brown, 2002 ▸) computed with the parameters of Brown & Altermatt (1985 ▸) are 1.89 valence units (v.u.) for Ca1 and 2.02 v.u. for Ca2, in good agreement with the expected value of 2. Likewise, the mean Ca—O bond length of 2.55 Å for Ca1 and 2.57 Å for Ca2 are in accord with the values for eight- and nine-coordinate Ca of 2.50 (15) and 2.56 (20) Å, respectively (Gagné & Hawthorne, 2016 ▸). Whereas the [Ca1O_8_] polyhedron is difficult to derive from a simple geometric figure, [Ca2O_9_] can be best described as a monocapped square anti­prism (Fig. 1 ▸).

All four Te atoms have an oxidation state of +IV and can be divided into two pairs with the most commonly observed three-coordination in the form of a trigonal pyramid (Te2 and Te4) and four-coordination in the form of a bis­phenoid (Te1 and Te3). The Te—O bond lengths within the [TeO_3_] trigonal pyramids are only slightly spread, ranging from 1.8522 (12) to 1.8994 (11) Å. The two [TeO_4_] bis­phenoids are characterised by two short bonds of < 2 Å and two longer bonds of > 2 Å, with the maximum at 2.3222 (12) Å for Te3. All Te—O bond lengths (Table 1 ▸) are in characteristic ranges for oxotellurates(IV) with three- and four-coordinate tellurium, as reviewed recently by Christy *et al.* (2016 ▸).

Bond valence sums for the four Te atoms computed with the parameters of Brese and O’Keeffe (1991 ▸) are 4.14, 4.07, 3.99 and 3.81 v.u., but are considerably lower when the revised parameters of Mills & Christy (2013 ▸) are used, *i.e.* 3.93, 3.80, 3.81 and 3.57 v.u.

**Figure 2:**
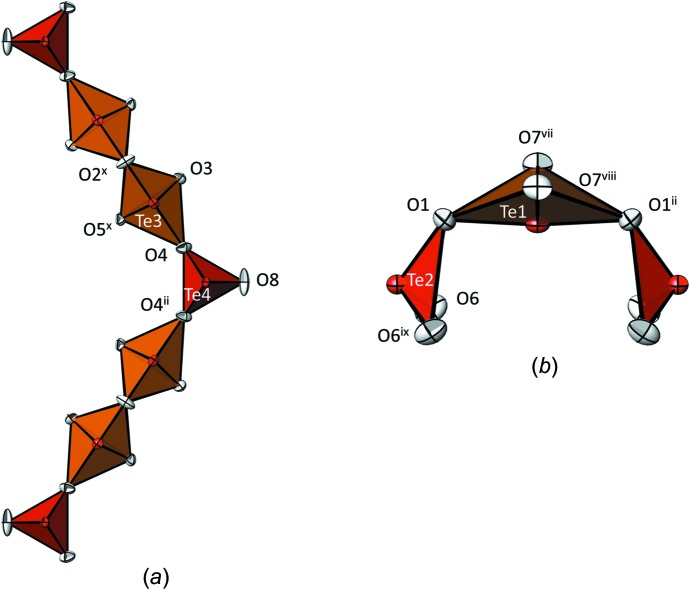
(*a*) The chain [Te_3_O_8_]^4−^ anion and (*b*) the finite [Te_3_O_8_]^4−^ anion in the crystal structure of Ca_2_Te_3_O_8_. Displacement ellipsoids are drawn at the 90% probability level. Symmetry codes refer to Table 1 ▸.

The oxotellurium(IV) network is built up from two different anions, both with composition [Te_3_O_8_]^4−^. One anion is made up from an infinite zigzag chain that extends parallel to [001] and consists of a pair of corner-sharing [Te3O_4_] bis­phenoids linked alternately to a [Te4O_3_] trigonal pyramid {= [(Te4O_1/1_O_2/2_)(Te3O_2/1_O_2/2_)_2_]_*n*_} (Fig. 2 ▸ *a*). The second oxotellurate(IV) anion is finite and is situated between neighbouring chain anions. It is comprised of a curved [Te_3_O_8_]^4−^ unit with a central Te1O_4_ bis­phenoid linked to two [Te2O_3_] trigonal pyramids {= [(Te1O_2/1_O_1/2_)_2_(Te2O_2/2_O_2/1_)]} (Fig. 2 ▸ *b*).

**Figure 3:**
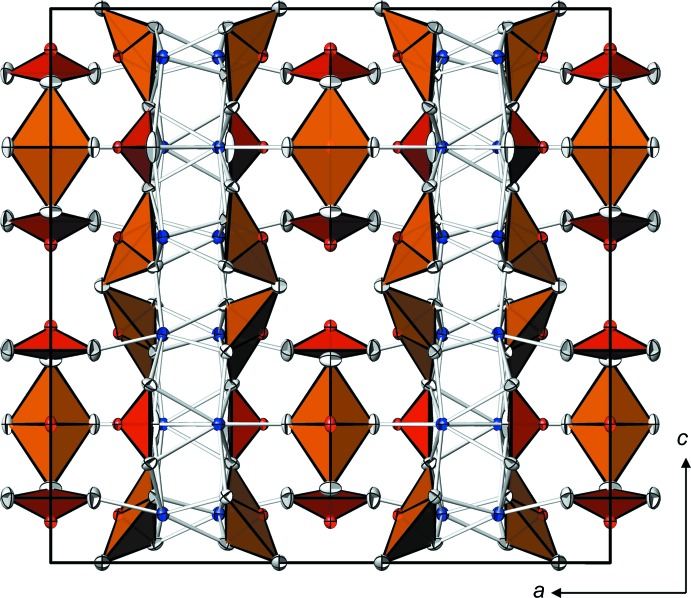
The crystal structure of Ca_2_Te_3_O_8_ in a projection along [0
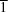
0]. Displacement ellipsoids and colour code as in Figs. 1 ▸ and 2 ▸.

In the crystal, the two types of [Te_3_O_8_]^4−^ anions are arranged in layers parallel to (100). Approximately at *x* ≃ 1/4 and 3/4, the calcium cations link adjacent layers into the three-dimensional framework (Fig. 3 ▸).

Ca_2_Te_3_O_8_ is isotypic with Pb_2_Te_3_O_8_ (Champarnaud-Mesjard *et al.*, 2001 ▸), but not with its higher alkaline earth homologue Sr_2_Te_3_O_8_, which is reported to have a different ortho­rhom­bic cell, with details of the structure not known (Elerman & Koçak, 1986 ▸). Comparison of the bond lengths of the [*M*O_*x*_] (*M* = Ca, Pb) polyhedra and the [TeO_3_] and [TeO_4_] units in the isotypic structures of Ca_2_Te_3_O_8_ and Pb_2_Te_3_O_8_ (Table 1 ▸) reveals nearly identical values for the individual oxotellurate(IV) units, but differences up to 0.6 Å for the metal–oxygen polyhedra. On one hand, this behaviour is ascribed to the different ionic radii for eight-coordinate Ca^II^ and Pb^II^ of 1.12 and 1.29 Å, respectively (Shannon, 1976 ▸), and, on the other hand, to the stereochemical activity (Galy *et al.*, 1975 ▸) of the 6*s* ^2^ free-electron lone pair located at Pb^II^ that is responsible for the formation of off-centred lead–oxygen polyhedra with either holo- or hemidirected oxygen ligands (Shimoni-Livny *et al.*, 1998 ▸).

For a qu­anti­tative structural comparison of the isotypic *M*_2_Te_3_O_8_ (*M* = Ca, Pb) structures, the program *compstru* (de la Flor *et al.*, 2016 ▸), available at the Bilbao Crystallographic Server (Aroyo *et al.*, 2006 ▸), was used. The degree of lattice distortion is 0.0205, the maximum distance between the atomic positions of paired atoms is 0.403 Å for pair O8, the arithmetic mean of all distances is 0.195 Å and the measure of similarity is 0.05.

## Synthesis and crystallization   

Crystals of Ca_2_Te_3_O_8_ were obtained as one of the products from a flux synthesis using a CsCl/NaCl salt mixture (molar ratio 0.65/0.35). To 1.5 g of the salt mixture were added CaO (0.075 g; freshly prepared by heating CaCO_3_ at 1473 K for 1 d) and TeO_2_ (0.425 g) according to a molar ratio of 1:2. The reaction mixture was placed in a silica ampoule that was subsequently evacuated and sealed. The ampoule was placed vertically in a furnace and heated from room temperature within 3 h to 793 K, kept at that temperature for 90 h and cooled within 10 h to room temperature. The silica ampoule was broken and the solidified melt leached out with water for two h. The colourless product was filtered off, washed with water and was dried in a stream of air. The title compound was present in the form of a few crystals that were distinguishable from the other crystals due to their characteristic square form (maximum edge length 1.5 mm). Other phases identified by single-crystal X-ray diffraction measurements of selected crystals and by powder X-ray diffraction measurements of the bulk were CaTe_2_O_5_ in the mica-like modification reported by Redman *et al.* (1970 ▸) as the main phase (tiny colourless plates) and Ca_4_Te_5_O_14_ (small colourless pinacoids; Weil, 2004 ▸).

## Refinement   

Crystal data, data collection and structure refinement details are summarized in Table 2 ▸. Starting coordinates for the refinement were taken from isotypic Pb_2_Te_3_O_8_ (Champarnaud-Mesjard *et al.*, 2001 ▸). Both remaining maximum and minimum electron-density peaks are located 0.64 and 0.30 Å from the Te2 site.

## Supplementary Material

Crystal structure: contains datablock(s) I, general. DOI: 10.1107/S2056989018017310/pk2611sup1.cif

Structure factors: contains datablock(s) I. DOI: 10.1107/S2056989018017310/pk2611Isup2.hkl

CCDC reference: 1883382

Additional supporting information:  crystallographic information; 3D view; checkCIF report

## Figures and Tables

**Table 1:** Comparison of bond lengths (Å) in Ca_2_Te_3_O_8_ and isotypic Pb_2_Te_3_O_8_

	**Ca_2_Te_3_O_8_** ^*a*^	**Pb_2_Te_3_O_8_** ^*b*^
*M*1—O3^i^	2.3198 (11)	2.372 (8)
*M*1—O6^ii^	2.3903 (13)	2.440 (8)
*M*1—O5	2.3924 (11)	2.470 (6)
*M*1—O3	2.4691 (12)	2.636 (8)
*M*1—O5^iii^	2.4712 (11)	2.934 (8)
*M*1—O3^iv^	2.6212 (12)	3.032 (8)
*M*1—O4	2.7186 (13)	
*M*1—O8	3.0360 (4)	3.069 (2)
*M*2—O8^v^	2.3089 (18)	2.439 (9)
*M*2—O7^vi^	2.3660 (17)	2.374 (10)
*M*2—O5^v^	2.4497 (11)	2.556 (6)
*M*2—O5^iii^	2.4497 (11)	2.556 (6)
*M*2—O8	2.4749 (19)	3.080 (11)
*M*2—O4^vi^	2.6335 (13)	2.732 (6)
*M*2—O4^iv^	2.6335 (13)	2.732 (6)
*M*2—O4	2.9154 (13)	3.342 (7)
*M*2—O4^ii^	2.9154 (13)	3.342 (7)
Te1—O7^vii^	1.8369 (17)	1.852 (10)
Te1—O7^viii^	1.8369 (17)	1.852 (10)
Te1—O1	2.1608 (17)	2.160 (9)
Te1—O1^ii^	2.1608 (17)	2.160 (9)
Te2—O6^ix^	1.8522 (12)	1.859 (8)
Te2—O6	1.8522 (12)	1.859 (8)
Te2—O1	1.8902 (17)	1.883 (10)
Te3—O3	1.8602 (11)	1.868 (8)
Te3—O5^*x*^	1.8743 (10)	1.856 (7)
Te3—O2^*x*^	2.0123 (5)	2.008 (3)
Te3—O4	2.3222 (12)	2.338 (6)
Te4—O8	1.8694 (17)	1.857 (10)
Te4—O4	1.8994 (11)	1.900 (7)
Te4—O4^ii^	1.8994 (11)	1.900 (7)

Notes: (*a*) this study; (*b*) Champarnaud-Mesjard *et al.* (2001 ▸); single-crystal data with *a* = 19.522 (4), *b* = 7.121 (1) and *c* = 18.813 (4) Å. Symmetry codes: (i) *x*, −*y* + 1, −*z* + 1; (ii) *x*, *y*, −*z* + 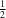; (iii) −*x* + 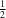, *y* + 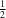, *z*; (iv) −*x* + 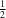, *y* − 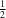, *z*; (v) −*x* + 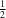, *y* + 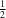, −*z* + 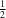; (vi) −*x* + 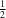, *y* − 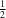, −*z* + 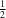; (vii) *x*, *y* − 1, *z*; (viii) −*x* + 1, *y* − 1, −*z* + 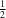; (ix) −*x* + 1, *y*, *z*; (*x*) *x*, *y* + 1, *z*.

**Table 2:** Experimental details

Crystal data	
Chemical formula	Ca_2_Te_3_O_8_
*M*_r_	590.95
Crystal system, space group	Orthorhombic, *C* *m* *c* *m*
Temperature (K)	297
*a*, *b*, *c* (Å)	18.7368 (15), 6.8399 (6), 18.5652 (15)
*V* (Å^3^)	2379.3 (3)
*Z*	12
Radiation type	Mo *K*α
μ (mm^−1^)	12.27
Crystal size (mm)	0.25 × 0.15 × 0.10

Data collection	
Diffractometer	Bruker APEXII CCD
Absorption correction	Multi-scan (*SADABS*; Krause *et al.*, 2015 ▸)
*T*_min_, *T*_max_	0.472, 0.750
No. of measured, independent and observed [*I* > 2σ(*I*)] reflections	47523, 6097, 5325
*R*_int_	0.043
(sin θ/λ)_max_ (Å^−1^)	1.057

Refinement	
*R*[*F* ^2^ > 2σ(*F* ^2^)], *wR*(*F* ^2^), *S*	0.023, 0.043, 1.12
No. of reflections	6097
No. of parameters	101
Δρ_max_, Δρ_min_ (e Å^−3^)	4.13, −2.08

Computer programs: *APEX3* and *SAINT* (Bruker, 2016 ▸), *SHELXL2017* (Sheldrick, 2015 ▸), *ATOMS* (Dowty, 2006 ▸) and *publCIF* (Westrip, 2010 ▸). Starting coordinates taken from an isotypic compound.

## supplementary crystallographic information

### Crystal data

**Table:**

Ca_2_Te_3_O_8_	*D*_x_ = 4.949 Mg m^−^^3^
*M**_r_* = 590.95	Mo *K*α radiation, λ = 0.71073 Å
Orthorhombic, *C**m**c**m*	Cell parameters from 9248 reflections
*a* = 18.7368 (15) Å	θ = 3.2–48.6°
*b* = 6.8399 (6) Å	µ = 12.27 mm^−^^1^
*c* = 18.5652 (15) Å	*T* = 297 K
*V* = 2379.3 (3) Å^3^	Plate, colourless
*Z* = 12	0.25 × 0.15 × 0.10 mm
*F*(000) = 3120	

### Data collection

**Table:**

Bruker APEXII CCD diffractometer	5325 reflections with *I* > 2σ(*I*)
ω scans	*R*_int_ = 0.043
Absorption correction: multi-scan (*SADABS*; Krause *et al.*, 2015)	θ_max_ = 48.7°, θ_min_ = 2.2°
*T*_min_ = 0.472, *T*_max_ = 0.750	*h* = −37→39
47523 measured reflections	*k* = −14→14
6097 independent reflections	*l* = −36→39

### Refinement

**Table:**

Refinement on *F*^2^	Primary atom site location: isomorphous structure methods
Least-squares matrix: full	*w* = 1/[σ^2^(*F*_o_^2^) + (0.0102*P*)^2^ + 6.3377*P*] where *P* = (*F*_o_^2^ + 2*F*_c_^2^)/3
*R*[*F*^2^ > 2σ(*F*^2^)] = 0.023	(Δ/σ)_max_ = 0.002
*wR*(*F*^2^) = 0.043	Δρ_max_ = 4.13 e Å^−^^3^
*S* = 1.12	Δρ_min_ = −2.08 e Å^−^^3^
6097 reflections	Extinction correction: SHELXL2017 (Sheldrick, 2015), Fc^*^=kFc[1+0.001xFc^2^λ^3^/sin(2θ)]^-1/4^
101 parameters	Extinction coefficient: 0.00143 (3)
0 restraints	

### Special details

**Table:**

Geometry. All esds (except the esd in the dihedral angle between two l.s. planes) are estimated using the full covariance matrix. The cell esds are taken into account individually in the estimation of esds in distances, angles and torsion angles; correlations between esds in cell parameters are only used when they are defined by crystal symmetry. An approximate (isotropic) treatment of cell esds is used for estimating esds involving l.s. planes.

### Fractional atomic coordinates and isotropic or equivalent isotropic displacement parameters (Å^2^)

**Table:**

	*x*	*y*	*z*	*U*_iso_*/*U*_eq_	
Ca1	0.29994 (2)	0.36584 (4)	0.41210 (2)	0.00884 (4)	
Ca2	0.19996 (2)	0.49754 (6)	0.250000	0.00819 (5)	
Te1	0.500000	0.08952 (3)	0.250000	0.00866 (3)	
Te2	0.500000	0.29743 (2)	0.07448 (2)	0.00865 (2)	
Te3	0.37898 (2)	0.84140 (2)	0.41362 (2)	0.00685 (2)	
Te4	0.37986 (2)	0.54478 (2)	0.250000	0.00616 (2)	
O1	0.500000	0.0728 (2)	0.13377 (9)	0.0150 (3)	
O2	0.40826 (9)	0.000000	0.500000	0.0146 (3)	
O3	0.31332 (6)	0.69421 (16)	0.46567 (6)	0.01057 (16)	
O4	0.32340 (7)	0.67071 (18)	0.32118 (6)	0.01226 (17)	
O5	0.31362 (6)	0.02866 (15)	0.38074 (6)	0.00918 (15)	
O6	0.42263 (7)	0.42092 (19)	0.11674 (8)	0.0165 (2)	
O7	0.42329 (9)	0.9223 (3)	0.250000	0.0135 (3)	
O8	0.31736 (10)	0.3318 (3)	0.250000	0.0209 (4)	

### Atomic displacement parameters (Å^2^)

**Table:**

	*U*^11^	*U*^22^	*U*^33^	*U*^12^	*U*^13^	*U*^23^
Ca1	0.00841 (9)	0.00807 (9)	0.01005 (9)	0.00102 (7)	−0.00031 (7)	0.00024 (7)
Ca2	0.00857 (13)	0.00778 (12)	0.00821 (12)	0.00113 (10)	0.000	0.000
Te1	0.00648 (6)	0.00643 (6)	0.01305 (7)	0.000	0.000	0.000
Te2	0.00773 (4)	0.00848 (4)	0.00974 (4)	0.000	0.000	−0.00001 (3)
Te3	0.00550 (3)	0.00710 (3)	0.00794 (3)	0.00067 (2)	0.00072 (2)	0.00095 (2)
Te4	0.00569 (4)	0.00572 (4)	0.00706 (4)	0.00012 (3)	0.000	0.000
O1	0.0227 (8)	0.0108 (6)	0.0116 (6)	0.000	0.000	0.0018 (5)
O2	0.0110 (6)	0.0209 (7)	0.0119 (6)	0.000	0.000	−0.0089 (5)
O3	0.0123 (4)	0.0103 (4)	0.0091 (4)	−0.0023 (3)	0.0028 (3)	0.0010 (3)
O4	0.0122 (4)	0.0157 (4)	0.0088 (4)	0.0031 (3)	0.0022 (3)	−0.0022 (3)
O5	0.0096 (4)	0.0072 (3)	0.0107 (4)	0.0021 (3)	−0.0006 (3)	0.0000 (3)
O6	0.0091 (4)	0.0175 (5)	0.0228 (6)	0.0037 (4)	0.0000 (4)	−0.0049 (4)
O7	0.0072 (5)	0.0142 (6)	0.0193 (7)	−0.0022 (5)	0.000	0.000
O8	0.0099 (7)	0.0077 (6)	0.0451 (12)	−0.0030 (5)	0.000	0.000

### Geometric parameters (Å, º)

**Table:**

Ca1—O3^i^	2.3198 (11)	Ca2—O4	2.9154 (13)
Ca1—O6^ii^	2.3903 (13)	Ca2—O4^ii^	2.9154 (13)
Ca1—O5	2.3924 (11)	Ca2—Te4	3.3863 (5)
Ca1—O3	2.4691 (12)	Ca2—Te4^vi^	3.4390 (5)
Ca1—O5^iii^	2.4712 (11)	Ca2—Te3^vi^	3.5434 (3)
Ca1—O3^iv^	2.6212 (12)	Te1—O7^vii^	1.8369 (17)
Ca1—O4	2.7186 (13)	Te1—O7^viii^	1.8369 (17)
Ca1—O8	3.0360 (4)	Te1—O1	2.1608 (17)
Ca1—Te3^iv^	3.3567 (4)	Te1—O1^ii^	2.1609 (17)
Ca1—Te3	3.5742 (4)	Te2—O6^ix^	1.8522 (12)
Ca1—Te4	3.5773 (4)	Te2—O6	1.8522 (12)
Ca1—Ca2	3.6575 (4)	Te2—O1	1.8902 (17)
Ca2—O8^v^	2.3089 (18)	Te3—O3	1.8602 (11)
Ca2—O7^vi^	2.3660 (17)	Te3—O5^x^	1.8743 (10)
Ca2—O5^v^	2.4497 (11)	Te3—O2^x^	2.0123 (5)
Ca2—O5^iii^	2.4497 (11)	Te3—O4	2.3222 (12)
Ca2—O8	2.4749 (19)	Te4—O8	1.8694 (17)
Ca2—O4^vi^	2.6335 (13)	Te4—O4	1.8994 (11)
Ca2—O4^iv^	2.6335 (13)	Te4—O4^ii^	1.8994 (11)
			
O3^i^—Ca1—O6^ii^	98.22 (5)	O8^v^—Ca2—Te3^vi^	103.87 (2)
O3^i^—Ca1—O5	93.20 (4)	O7^vi^—Ca2—Te3^vi^	61.771 (12)
O6^ii^—Ca1—O5	89.68 (4)	O5^v^—Ca2—Te3^vi^	29.94 (2)
O3^i^—Ca1—O3	75.88 (4)	O5^iii^—Ca2—Te3^vi^	146.33 (3)
O6^ii^—Ca1—O3	81.32 (4)	O8—Ca2—Te3^vi^	103.47 (2)
O5—Ca1—O3	164.59 (4)	O4^vi^—Ca2—Te3^vi^	40.94 (3)
O3^i^—Ca1—O5^iii^	113.79 (4)	O4^iv^—Ca2—Te3^vi^	96.03 (3)
O6^ii^—Ca1—O5^iii^	134.77 (4)	O4—Ca2—Te3^vi^	147.38 (2)
O5—Ca1—O5^iii^	117.99 (4)	O4^ii^—Ca2—Te3^vi^	93.73 (2)
O3—Ca1—O5^iii^	76.84 (4)	Te4—Ca2—Te3^vi^	116.377 (7)
O3^i^—Ca1—O3^iv^	68.72 (4)	Te4^vi^—Ca2—Te3^vi^	63.068 (6)
O6^ii^—Ca1—O3^iv^	159.11 (4)	O7^vii^—Te1—O7^viii^	102.97 (11)
O5—Ca1—O3^iv^	75.37 (4)	O7^vii^—Te1—O1	88.11 (3)
O3—Ca1—O3^iv^	109.69 (4)	O7^viii^—Te1—O1	88.11 (3)
O5^iii^—Ca1—O3^iv^	66.05 (4)	O7^vii^—Te1—O1^ii^	88.11 (3)
O3^i^—Ca1—O4	136.52 (4)	O7^viii^—Te1—O1^ii^	88.11 (3)
O6^ii^—Ca1—O4	65.45 (4)	O1—Te1—O1^ii^	173.92 (9)
O5—Ca1—O4	124.82 (4)	O7^vii^—Te1—Ca2^vi^	28.98 (5)
O3—Ca1—O4	62.35 (4)	O7^viii^—Te1—Ca2^vi^	131.95 (6)
O5^iii^—Ca1—O4	69.34 (4)	O1—Te1—Ca2^vi^	89.497 (8)
O3^iv^—Ca1—O4	135.24 (4)	O1^ii^—Te1—Ca2^vi^	89.497 (8)
O3^i^—Ca1—O8	160.81 (5)	O7^vii^—Te1—Ca2^xi^	131.95 (6)
O6^ii^—Ca1—O8	71.74 (5)	O7^viii^—Te1—Ca2^xi^	28.98 (5)
O5—Ca1—O8	70.93 (4)	O1—Te1—Ca2^xi^	89.497 (8)
O3—Ca1—O8	117.27 (4)	O1^ii^—Te1—Ca2^xi^	89.497 (8)
O5^iii^—Ca1—O8	83.89 (4)	Ca2^vi^—Te1—Ca2^xi^	160.934 (13)
O3^iv^—Ca1—O8	115.39 (4)	O6^ix^—Te2—O6	103.01 (8)
O4—Ca1—O8	54.98 (4)	O6^ix^—Te2—O1	97.12 (6)
O3^i^—Ca1—Te3^iv^	95.22 (3)	O6—Te2—O1	97.12 (6)
O6^ii^—Ca1—Te3^iv^	165.99 (3)	O6^ix^—Te2—Ca1^xii^	30.77 (4)
O5—Ca1—Te3^iv^	93.50 (3)	O6—Te2—Ca1^xii^	133.69 (4)
O3—Ca1—Te3^iv^	98.24 (3)	O1—Te2—Ca1^xii^	93.563 (8)
O5^iii^—Ca1—Te3^iv^	33.33 (2)	O6^ix^—Te2—Ca1^ii^	133.69 (4)
O3^iv^—Ca1—Te3^iv^	33.48 (2)	O6—Te2—Ca1^ii^	30.77 (4)
O4—Ca1—Te3^iv^	101.83 (3)	O1—Te2—Ca1^ii^	93.563 (8)
O8—Ca1—Te3^iv^	96.42 (4)	Ca1^xii^—Te2—Ca1^ii^	163.902 (10)
O3^i^—Ca1—Te3	96.23 (3)	O3—Te3—O5^x^	96.14 (5)
O6^ii^—Ca1—Te3	57.30 (3)	O3—Te3—O2^x^	93.33 (5)
O5—Ca1—Te3	146.60 (3)	O5^x^—Te3—O2^x^	93.97 (5)
O3—Ca1—Te3	29.18 (3)	O3—Te3—O4	79.35 (5)
O5^iii^—Ca1—Te3	87.05 (3)	O5^x^—Te3—O4	79.04 (5)
O3^iv^—Ca1—Te3	137.74 (3)	O2^x^—Te3—O4	169.18 (6)
O4—Ca1—Te3	40.52 (3)	O3—Te3—Ca1^iii^	51.01 (4)
O8—Ca1—Te3	91.90 (3)	O5^x^—Te3—Ca1^iii^	46.42 (3)
Te3^iv^—Ca1—Te3	117.328 (9)	O2^x^—Te3—Ca1^iii^	104.60 (5)
O3^i^—Ca1—Te4	146.96 (3)	O4—Te3—Ca1^iii^	64.59 (3)
O6^ii^—Ca1—Te4	49.84 (3)	O3—Te3—Ca2^v^	109.41 (4)
O5—Ca1—Te4	94.60 (3)	O5^x^—Te3—Ca2^v^	40.72 (3)
O3—Ca1—Te4	89.14 (3)	O2^x^—Te3—Ca2^v^	129.382 (18)
O5^iii^—Ca1—Te4	90.46 (3)	O4—Te3—Ca2^v^	47.99 (3)
O3^iv^—Ca1—Te4	144.22 (3)	Ca1^iii^—Te3—Ca2^v^	63.955 (8)
O4—Ca1—Te4	31.52 (2)	O3—Te3—Ca1	40.32 (4)
O8—Ca1—Te4	31.50 (3)	O5^x^—Te3—Ca1	110.40 (3)
Te3^iv^—Ca1—Te4	116.244 (9)	O2^x^—Te3—Ca1	127.580 (17)
Te3—Ca1—Te4	61.433 (6)	O4—Te3—Ca1	49.52 (3)
O3^i^—Ca1—Ca2	154.68 (3)	Ca1^iii^—Te3—Ca1	68.372 (6)
O6^ii^—Ca1—Ca2	105.63 (3)	Ca2^v^—Te3—Ca1	95.427 (9)
O5—Ca1—Ca2	95.28 (3)	O3—Te3—Ca1^i^	26.53 (4)
O3—Ca1—Ca2	99.17 (3)	O5^x^—Te3—Ca1^i^	105.99 (3)
O5^iii^—Ca1—Ca2	41.77 (3)	O2^x^—Te3—Ca1^i^	68.39 (3)
O3^iv^—Ca1—Ca2	90.45 (3)	O4—Te3—Ca1^i^	105.36 (3)
O4—Ca1—Ca2	51.91 (3)	Ca1^iii^—Te3—Ca1^i^	68.861 (8)
O8—Ca1—Ca2	42.13 (4)	Ca2^v^—Te3—Ca1^i^	132.402 (9)
Te3^iv^—Ca1—Ca2	60.505 (8)	Ca1—Te3—Ca1^i^	60.638 (8)
Te3—Ca1—Ca2	89.690 (9)	O8—Te4—O4	90.26 (6)
Te4—Ca1—Ca2	55.803 (9)	O8—Te4—O4^ii^	90.26 (6)
O8^v^—Ca2—O7^vi^	94.49 (6)	O4—Te4—O4^ii^	88.18 (7)
O8^v^—Ca2—O5^v^	84.22 (3)	O8—Te4—Ca2	45.74 (6)
O7^vi^—Ca2—O5^v^	85.27 (3)	O4—Te4—Ca2	59.26 (4)
O8^v^—Ca2—O5^iii^	84.22 (3)	O4^ii^—Te4—Ca2	59.26 (4)
O7^vi^—Ca2—O5^iii^	85.27 (3)	O8—Te4—Ca2^v^	115.43 (6)
O5^v^—Ca2—O5^iii^	164.44 (5)	O4—Te4—Ca2^v^	49.41 (4)
O8^v^—Ca2—O8	125.35 (5)	O4^ii^—Te4—Ca2^v^	49.42 (4)
O7^vi^—Ca2—O8	140.16 (6)	Ca2—Te4—Ca2^v^	69.699 (8)
O5^v^—Ca2—O8	97.59 (3)	O8—Te4—Ca1	58.067 (9)
O5^iii^—Ca2—O8	97.59 (3)	O4—Te4—Ca1	48.45 (4)
O8^v^—Ca2—O4^vi^	144.79 (4)	O4^ii^—Te4—Ca1	120.49 (4)
O7^vi^—Ca2—O4^vi^	69.70 (5)	Ca2—Te4—Ca1	63.297 (6)
O5^v^—Ca2—O4^vi^	63.85 (3)	Ca2^v^—Te4—Ca1	97.242 (7)
O5^iii^—Ca2—O4^vi^	123.62 (4)	O8—Te4—Ca1^ii^	58.067 (9)
O8—Ca2—O4^vi^	76.04 (5)	O4—Te4—Ca1^ii^	120.49 (4)
O8^v^—Ca2—O4^iv^	144.79 (4)	O4^ii^—Te4—Ca1^ii^	48.45 (4)
O7^vi^—Ca2—O4^iv^	69.70 (5)	Ca2—Te4—Ca1^ii^	63.299 (6)
O5^v^—Ca2—O4^iv^	123.62 (4)	Ca2^v^—Te4—Ca1^ii^	97.242 (7)
O5^iii^—Ca2—O4^iv^	63.85 (3)	Ca1—Te4—Ca1^ii^	114.545 (11)
O8—Ca2—O4^iv^	76.04 (5)	Te2—O1—Te1	122.58 (9)
O4^vi^—Ca2—O4^iv^	60.24 (5)	Te3^vii^—O2—Te3^i^	148.36 (9)
O8^v^—Ca2—O4	73.10 (5)	Te3—O3—Ca1^i^	132.49 (6)
O7^vi^—Ca2—O4	149.63 (3)	Te3—O3—Ca1	110.51 (5)
O5^v^—Ca2—O4	119.75 (4)	Ca1^i^—O3—Ca1	102.82 (4)
O5^iii^—Ca2—O4	66.29 (3)	Te3—O3—Ca1^iii^	95.51 (5)
O8—Ca2—O4	58.73 (4)	Ca1^i^—O3—Ca1^iii^	111.28 (4)
O4^vi^—Ca2—O4	134.77 (3)	Ca1—O3—Ca1^iii^	99.92 (4)
O4^iv^—Ca2—O4	104.43 (4)	Te4—O4—Te3	119.50 (6)
O8^v^—Ca2—O4^ii^	73.10 (5)	Te4—O4—Ca2^v^	97.37 (5)
O7^vi^—Ca2—O4^ii^	149.63 (3)	Te3—O4—Ca2^v^	91.07 (4)
O5^v^—Ca2—O4^ii^	66.29 (3)	Te4—O4—Ca1	100.03 (5)
O5^iii^—Ca2—O4^ii^	119.75 (4)	Te3—O4—Ca1	89.96 (4)
O8—Ca2—O4^ii^	58.73 (4)	Ca2^v^—O4—Ca1	159.36 (5)
O4^vi^—Ca2—O4^ii^	104.43 (4)	Te4—O4—Ca2	86.69 (4)
O4^iv^—Ca2—O4^ii^	134.77 (3)	Te3—O4—Ca2	153.51 (5)
O4—Ca2—O4^ii^	53.91 (5)	Ca2^v^—O4—Ca2	89.17 (4)
O8^v^—Ca2—Te4	92.60 (5)	Ca1—O4—Ca2	80.88 (3)
O7^vi^—Ca2—Te4	172.91 (4)	Te3^vii^—O5—Ca1	130.51 (5)
O5^v^—Ca2—Te4	95.46 (3)	Te3^vii^—O5—Ca2^vi^	109.34 (5)
O5^iii^—Ca2—Te4	95.46 (3)	Ca1—O5—Ca2^vi^	108.29 (4)
O8—Ca2—Te4	32.75 (4)	Te3^vii^—O5—Ca1^iv^	100.25 (5)
O4^vi^—Ca2—Te4	104.27 (3)	Ca1—O5—Ca1^iv^	106.55 (4)
O4^iv^—Ca2—Te4	104.27 (3)	Ca2^vi^—O5—Ca1^iv^	96.02 (4)
O4—Ca2—Te4	34.05 (2)	Te2—O6—Ca1^ii^	125.87 (7)
O4^ii^—Ca2—Te4	34.05 (2)	Te1^x^—O7—Ca2^v^	128.92 (9)
O8^v^—Ca2—Te4^vi^	146.15 (5)	Te4—O8—Ca2^vi^	149.29 (10)
O7^vi^—Ca2—Te4^vi^	51.66 (4)	Te4—O8—Ca2	101.52 (8)
O5^v^—Ca2—Te4^vi^	91.90 (3)	Ca2^vi^—O8—Ca2	109.19 (7)
O5^iii^—Ca2—Te4^vi^	91.90 (3)	Te4—O8—Ca1	90.43 (3)
O8—Ca2—Te4^vi^	88.51 (4)	Ca2^vi^—O8—Ca1	93.49 (3)
O4^vi^—Ca2—Te4^vi^	33.21 (2)	Ca2—O8—Ca1	82.49 (4)
O4^iv^—Ca2—Te4^vi^	33.21 (2)	Te4—O8—Ca1^ii^	90.43 (3)
O4—Ca2—Te4^vi^	135.31 (3)	Ca2^vi^—O8—Ca1^ii^	93.49 (3)
O4^ii^—Ca2—Te4^vi^	135.31 (3)	Ca2—O8—Ca1^ii^	82.49 (4)
Te4—Ca2—Te4^vi^	121.252 (12)	Ca1—O8—Ca1^ii^	164.82 (7)

Symmetry codes: (i) *x*, −*y*+1, −*z*+1; (ii) *x*, *y*, −*z*+1/2; (iii) −*x*+1/2, *y*+1/2, *z*; (iv) −*x*+1/2, *y*−1/2, *z*; (v) −*x*+1/2, *y*+1/2, −*z*+1/2; (vi) −*x*+1/2, *y*−1/2, −*z*+1/2; (vii) *x*, *y*−1, *z*; (viii) −*x*+1, *y*−1, −*z*+1/2; (ix) −*x*+1, *y*, *z*; (x) *x*, *y*+1, *z*; (xi) *x*+1/2, *y*−1/2, *z*; (xii) −*x*+1, *y*, −*z*+1/2.

## References

[bb1] Aroyo, M. I., Perez-Mato, J. M., Capillas, C., Kroumova, E., Ivantchev, S., Madariaga, G., Kirov, A. & Wondratschek, H. (2006). *Z. Kristallogr.* **221**, 15–27.

[bb2] Barrier, N., Rueff, J. M., Lepetit, M. B., Contreras-Garcia, J., Malo, S. & Raveau, B. (2009). *Solid State Sci.* **11**, 289–293.

[bb3] Brese, N. E. & O’Keeffe, M. (1991). *Acta Cryst.* B**47**, 192–197.

[bb4] Brown, I. D. (2002). In *The Chemical Bond in Inorganic Chemistry: The Bond Valence Model*. Oxford University Press.

[bb5] Brown, I. D. & Altermatt, D. (1985). *Acta Cryst.* B**41**, 244–247.

[bb6] Bruker (2016). *APEX3* and *SAINT*. Bruker AXS Inc., Madison, Wisconsin, USA.

[bb7] Champarnaud-Mesjard, J. C., Thomas, P., Colas-Dutreilh, M. & Oufkir, A. (2001). *Z. Kristallogr. New Cryst. Struct.* **216**, 185–186.

[bb8] Christy, A. G., Mills, S. J. & Kampf, A. R. (2016). *Mineral. Mag.* **80**, 415–545.

[bb9] Dowty, E. (2006). *ATOMS for Windows*. Shape Software, 521 Hidden Valley Road, Kingsport, TN 37663, USA.

[bb10] Elerman, Y. & Koçak, M. (1986). *J. Appl. Cryst.* **19**, 410.

[bb11] Flor, G. de la, Orobengoa, D., Tasci, E., Perez-Mato, J. M. & Aroyo, M. I. (2016). *J. Appl. Cryst.* **49**, 653–664.

[bb12] Gagné, O. C. & Hawthorne, F. C. (2016). *Acta Cryst.* B**72**, 602–625. 10.1107/S2052520616008507 PMC4971548 27484381

[bb13] Galy, J., Meunier, G., Anderson, S. & Åström, A. (1975). *J. Solid State Chem.* **13**, 142–159.

[bb14] Krause, L., Herbst-Irmer, R., Sheldrick, G. M. & Stalke, D. (2015). *J. Appl. Cryst.* **48**, 3–10. 10.1107/S1600576714022985 PMC4453166 26089746

[bb15] Mills, S. J. & Christy, A. G. (2013). *Acta Cryst.* B**69**, 145–149. 10.1107/S2052519213004272 23719701

[bb16] Mishra, R., Namboodiri, P. N., Tripathi, S. N. & Dharwadkar, S. R. (1998). *J. Alloys Compd.* **280**, 56–64.

[bb17] Poupon, M., Barrier, N., Petit, S., Clevers, S. & Dupray, V. (2015). *Inorg. Chem.* **54**, 5660–5670. 10.1021/acs.inorgchem.5b00037 26035739

[bb18] Redman, M. J., Chen, J. H., Binnie, W. P. & Mallo, W. J. (1970). *J. Am. Chem. Soc.* **53**, 645–648.

[bb19] Shannon, R. D. (1976). *Acta Cryst.* A**32**, 751–767.

[bb20] Sheldrick, G. M. (2015). *Acta Cryst.* A**71**, 3–8.

[bb21] Shimoni-Livny, L., Glusker, J. P. & Bock, C. W. (1998). *Inorg. Chem.* **37**, 1853–1867.

[bb22] Stöger, B., Weil, M., Zobetz, E. & Giester, G. (2009). *Acta Cryst.* B**65**, 167–181. 10.1107/S0108768109002997 19299873

[bb23] Tripathi, S. N., Mishra, R., Mathews, M. D. & Namboodiri, P. N. (2001). *Powder Diffr.* **16**, 205–211.

[bb24] Weil, M. (2004). *Solid State Sci.* **6**, 29–37.

[bb25] Weil, M., Heymann, G. & Huppertz, H. (2016). *Eur. J. Inorg. Chem.* pp. 2374–3579.

[bb26] Weil, M. & Stöger, B. (2008). *Acta Cryst.* C**64**, i79–i81. 10.1107/S0108270108024827 18758006

[bb27] Westrip, S. P. (2010). *J. Appl. Cryst.* **43**, 920–925.

